# Moving beyond 'rates, roads and rubbish': How do local governments make choices about healthy public policy to prevent obesity?

**DOI:** 10.1186/1743-8462-6-20

**Published:** 2009-08-23

**Authors:** Steven Allender, Erin Gleeson, Brad Crammond, Gary Sacks, Mark Lawrence, Anna Peeters, Bebe Loff, Boyd Swinburn

**Affiliations:** 1 Department of Public Health, University of Oxford, Oxford, UK; 2 WHO Collaborating Centre on Obesity, Deakin University, Melbourne, Australia; 3 School of Public Health and Preventive Medicine, Monash University, Melbourne, Australia

## Abstract

While the causes of obesity are well known traditional education and treatment strategies do not appear to be making an impact. One solution as part of a broader complimentary set of strategies may be regulatory intervention at local government level to create environments for healthy nutrition and increased physical activity. Semi structured interviews were conducted with representatives of local government in Australia. Factors most likely to facilitate policy change were those supported by external funding, developed from an evidence base and sensitive to community and market forces. Barriers to change included a perceived or real lack of power to make change and the complexity of the legislative framework. The development of a systematic evidence base to provide clear feedback on the size and scope of the obesity epidemic at a local level, coupled with cost benefit analysis for any potential regulatory intervention, are crucial to developing a regulatory environment which creates the physical and social environment required to prevent obesity.

## Introduction

Obesity is a major risk factor in the development of non-communicable diseases such as Type II diabetes, coronary heart disease and many cancers [1,2]. For countries like England overweight and obesity can be attributed to more than 65,000 deaths and around 5% of total NHS expenditure (more than £3 billion annually) [3]. In Australia obesity rates are increasing among children and disproportionately among people from socially and economically disadvantaged backgrounds [4-9]. The increase in obesity prevalence is due largely to increased consumption of high energy density foods, very low consumption of fruit and vegetables and a shift to less active transport and more sedentary leisure time activities [10-12].

Current obesity trends suggest that existing education and treatment strategies alone are not potent or sustainable enough to stem the obesity epidemic and that environmental change will certainly be needed [13]. Areas of low walkability [14], a high density of fast food outlets [15], and the cheap price of energy dense foods [16], have been identified as environmental factors contributing to the obesity epidemic. Alterations to the policy and regulatory environment represents one method of driving changes in the physical, economic, and socio-cultural environments [17].

### Regulation and obesity prevention

Internationally there are a number of regulations which, while enacted for other reasons, contribute to obesity prevention. Examples include bans on TV advertisements targeting children, nutrition information panels on food and speed restrictions in neighbourhoods (protecting pedestrians and cyclists from injuries). There has been much discussion about regulatory reform options to specifically address obesity [18,19], including at a global level [20], but there has been little research and piecemeal action in the area. The NSW Healthy Canteen Strategy [21] and the Queensland Healthy Food and Drink Supply for Schools [22] are Australian examples of specific regulatory response to childhood obesity. It might be argued, however, that until a programme of complementary, society-wide law reform is implemented, little change can be expected in the overwhelming trend towards obesity. As has occurred for smoking, road injuries, and many infectious disease epidemics, a strong regulatory environment, as one of several parallel complementary strategies, provides the foundation for long term cultural and attitudinal changes towards health promoting behaviours.

Any intervention involving law reform towards obesity prevention must be sensitive to the great complexities of the regulatory environment involved. Australia has a three tiered Federal/State/Local system, each of which has different regulatory responsibilities. At the third tier local governments take responsibility for sanitation, pest control and food safety. Within this remit there is limited direct responsibility relevant to obesity. This has led to the common misconception that local government is concerned solely with '*roads, rates and rubbish'*. However, in Victoria, the Victorian *Health Act 1958*, requires local governments prepare Municipal Public Health Plans, which councils use to set the health policies and strategies for each municipality. Local government in Victoria also has responsibilities under the *Planning and Environment Act 1987 *to regulate the built environment in new and existing suburbs. In addition local governments can make changes to improve the cycling and walking environment, the land-use mix, and the provision of open spaces for physical activity. This responsibility is formally recognised by the Victorian Department of Human Services which recommends that local government include these elements in the compulsory Municipal Public Health Plans. The Victorian State government report on the MPHP Framework suggests that since 2001 the plan has had some positive impact on local government planning and that the principles of the plan have been incorporated into the majority of MPHPs. The Framework has also been adopted by a range of other state government areas including Municipal Early Years Plans, Neighbourhood Renewal, and Emergency Management[23]

Within the complexity of the Federal/State/Local government system we set out to understand the ways in which the regulatory environment may support healthy nutrition and physical activity. In this paper we have narrowed our focus to local government in Victoria because of the potential for regulatory intervention at this level to prevent obesity. We were interested in the barriers and facilitators local councils face when attempting policy and regulatory changes that may reduce obesity. This study was informed by the following question:

What are the barriers and facilitators to local government policy change in relation to environments for healthy eating and physical activity?

## Methods

We were interested in the lived experience of a small number of people working within the policy and regulatory framework for nutrition and physical activity at a local government level. To this end a qualitative approach was deemed the most appropriate, in which in depth data were collected and analysed relating to a few cases, allowing for the development of deeper understanding of the particular phenomena under study and the development of greater theoretical understanding.

We took a social constructionist stance to this study [24], in which the experience of each interview participant is understood to vary depending on their own understanding of the phenomena under study as well as their current social environment and their previous experiences. An interpretive, phenomenological approach to data collection was considered appropriate within a research process that aimed to elicit participants' experience of a particular phenomenon (in this case the policy and regulatory framework pertaining to obesity, nutrition and physical activity).

### Recruitment

A key informant snowball sampling technique was used to identify potential participants in this study. Initial discussions with experts in local government indicated that each local council was quite different in terms of structure, history and priorities. As such, the views of people who have experience working within and on behalf of more than one council and who have worked with or in local government over long periods of time represented a good starting point. Following initial discussions with an expert in urban planning and local government a number of key people were identified. These key informants were approached to participate in the study and during the course of an interview they were asked to identify others they felt may be able to provide insight into our research questions. These people were subsequently approached based on the recommendation of initial participants to participate in the study, and so on. In addition we approached a number of councils to include those with day to day experience of trying to achieve policy change.

### Participants

We continued to recruit participants until data saturation had occurred; the point at which no new insights were being derived from interview [25]. Subsequently eleven participants completed semi-structured interviews. Six participants were employees of three local councils in Victoria, two from rural Victoria and one from metropolitan Melbourne. Two participants were employees of local government representative organisations, and three were urban and social planners. The majority of the participants had previous experience in many roles working in numerous different local councils. Participants included a programme coordinator, a strategic manager, a strategic planner, a Chief Executive Officer, two programme coordinators, an urban planner, a social planner and a strategic manager.

### Data collection

Previous work with policy academics and practitioners identified nine separate policy areas in which to promote healthy nutrition and increased physical activity. These areas were the walking environment, cycling environment, land use mix, public liability, the built environment for physical activity, open spaces for physical activity, food policy and billboards and signage. Documents were drafted to demonstrate potential regulatory interventions in each area. These policy documents were used as part of a semi structured interview schema to provide the basis for each interview. The schedule included questions on the role of the participant, their experience with local government health policy, and physical activity, nutrition and obesity policy at local government level. A semi structured approach provides the flexibility to follow up elements of the conversation which may not sit within the interview schema but which may provide further insight to the research question. Interviews were recorded using a digital voice recorder and later transcribed. Interview participants were given the opportunity to review and edit their transcripts for accuracy.

### Analysis

Data were analysed using the constant comparative method [25] which is based on grounded theory and begins with open inductive coding involving line-by-line reading of interview transcripts. Immediately following each interview and during the analysis process each reviewer noted down emerging understandings in the form of research memos. Emerging findings were constantly compared with the existing data to check and confirm intermediate conclusions while simultaneously informing the subsequent interview schedule.

Data collection and initial stages of analysis were undertaken simultaneously and subsequent interviews reflected the understanding developed from preliminary analysis of previous interviews. Transcripts were checked against the initial recording of each interview and key topics were noted. Transcripts were entered into NVIVO 7 for analysis. Clean transcripts were independently reviewed and coded by three researchers (SA, EG and BC). The researchers conferred where there was disagreement over coding of texts and codes were agreed upon.

### Ethics

Ethical approval was granted for this study by the committees of Deakin (EC 232-2007) and Monash Universities.

## Results

The results section presents excerpts from participants which summarise the themes of the interviews. In the first instance we examined what factors facilitated or discouraged policy change within local government. The changes most likely to improve the environment for healthy eating and physical activity within local areas were those supported by external funding, developed from a local evidence base and sensitive to community and market forces. Barriers to change at the local government level included a perceived or real lack of power to make change, the complexity of the legislative framework and a reluctance to increase regulation in what was already considered to be a heavily regulated environment.

### Evidence of local problems and effectiveness research

Participants described the importance of evidence in supporting policy change within local government. A number of participants recalled examples from their own experience when research evidence had driven health policy change. This Strategic Manager used an example of how local social inequalities data led to policy change within their local government:

Evidence base is really important. We start a project because it's either come out of a previous piece of research we've done and we've identified a question that's not yet answered, or Census data comes out and we're saying there's increasing issues between rich and poor, separation of rich and poor...

The importance of supporting policy change from a strong evidence base was consistent across interviews. A programme coordinator from another council described how the analysis of body mass index (BMI) data provided the impetus for the development of obesity prevention programmes:

*...we have collected data through our maternal and child health nurses... 25% of our four to five year old population is obese, we're more obese than we are overweight, if that makes sense... So now that's a red flag for us, so now it's one of our health priorities in my department*.

Evidence is also critical in gaining support from other members of local councils for proposed policy change. A Team Leader provided a succinct summary of the situation in their local council:

*If you haven't got the data ... with what you're proposing, then it's not going to be seen as a valuable inclusion into planning or policy. So it's really crucial and I think that's why a lot of us do a lot of research and data around what we're working on and strategies and plans to justify and support what we're trying to do*.

For local government 'evidence' means more than statistics about disease prevalence or incidence. While traditional prevalence data can put a health issue on the agenda, cost benefit data can support a change in policy direction. One Strategic Manager commented:

*Look sometimes it's reactionary, when stats come out at a state level, the increase in obesity and diabetes, they are reactionary so that they'll start thinking right we need to do this at a local level... it needs to be in the dollar value of how it's actually going to save them money*.

### Provision of funding

The provision of funding from the State Government has substantial influence on policy priorities at a local level. A Research and Policy officer described how State funding for public transport has a flow on effect at a local level:

*In terms of bike and pedestrian paths, sometimes they'll get the area that runs public transport and Vic Roads will sometimes have grants for bike paths and so it will be a case of saying, Council might develop up a strategy to say "this is our plan, this is where we want all our bike paths and this is what we want" and they might say, "we want to put in this amount of money" and the State Department might have a grant program and they'll say, "ok well we'll match you or we'll put in this amount of money" and so they can fund their bike paths*.

However, programmes initiated in response to external funding can suffer longer term sustainability problems at local government level. External funding is often only allocated over relatively short periods of time and local government are faced with the challenge of demonstrating a new initiative is effective and can be sustainable. One programme coordinator described the process:

*... if it's externally funded it takes a long time for policy to be enacted and if at that time policies are starting to finally get some movement behind them and then funding is pulled. It's that externally funded programs that are advocating for policy can be a very good thing but then they can be a bad thing because the momentum can be lost... or the money runs out and you hope that it becomes sustainable but it doesn't always happen*.

A number of participants introduced the term "cost shifting"; a commonly cited barrier to local councils taking on policy change. "Cost shifting" describes a process whereby State or Federal Governments provide seed funding for policy change and then remove the funding once the programme is established with the expectation that local government will find the funding for the policy to continue. One Manager from a representative body used State funding of maternal and child health policy as an example:

*When maternal and child health started it was funded 75% by the state and councils were the beneficiaries of that funding to help deliver a service, now it's at about 20% state funding. And the services are quite prescribed by the state in terms of what they can do and of course the community's expectations here and councils have responded to what the community expects*.

Councils in affluent areas of the state can charge higher rates than councils with low socio economic status (and especially rural areas) with huge ramifications for council resources. Victoria has been in the grip of a drought for the past five years which has rendered many open recreation spaces and sports playing surfaces unusable. As one member of a local government representative body noted the more affluent councils are able to use their social advantage to overcome these problems and create environments that support physical activity despite the drought:

*Because in [affluent inner city council] the rates are higher, they can have a well serviced public. [Another affluent inner city council] basically buy water and keep their parks irrigated*.

### Council structure and support

A third theme which emerged from interviews was the vital role of the structure and leadership within the council plays in health policy change. Councils with better communication between departments seem to be better able to provide policy responses to health problems. Across council approaches were deemed important in avoiding frustration and confusion over conflicting aims, duplication of effort and wasted resources. One manager from a local council described it as follows:

*I think you'll find a lot of other local government who will be frustrated... because they haven't been successful with joined up thinking. You've got to have your social planner talking to your land use planners and particularly talking to your capital works people. I often hear social planners say, 'It's the town planners fault...' It's often not the town planners that are actually making the changes, its engineers and the asset design people that can change public areas*.

A structure that allows sharing of different ideas between the different arms of local government can be more powerful in effecting healthy policy change. One programme coordinator highlighted the differences in priorities between departments within local councils:

*And a good example of that is across our engineers who are technical minded people and trying to work with them with a roundabout and safety school crossings. We're thinking from a totally different perspective than they are. We're trying to make sure that it's useable and it's safe for kids to cross the road and they're trying to make sure it's useable for the traffic*.

Strong leadership with a clear understanding and interest in proactive health policy is more likely to support changes towards obesity prevention. The importance of a strong CEO with a clear understanding of the potential for policy to improve health is described by one strategic manager:

*If you have a CEO coming through from like a community services sort of area, there's more likelihood that that council will be much more active in intervening in health issues, social issues. If it's an engineer, it's more likely that they are going to be focused on asset management issues, infrastructure and those sorts of things*.

### Lobbying from within community

Community wishes could override evidence based decisions supported by cost benefit analysis and external funding. A Strategic Planner within one local council described how community action can be more powerful in leading policy change than the evidence base for local government:

*If you go back 10 years there was sort of some toes in the water about recycling. The community sort of took to it with almost religious fervour and councils had to do it, you know, it was not something they couldn't not do, even though there was a whole heap of evidence that said..., we would've been much better putting all that resource and energy into reducing use in the first place rather than recycling*.

### State legislation

Local government powers are defined primarily by State Government legislation. Consequently local governments are dependent upon the State for their power to enact policy and State laws overrule any created by local government. One social planner notes the importance of State legislation to the planning scheme, stating:

*From where I sit if it's not in the [State] Planning and Environment Act it doesn't have to happen*.

Another major part of the State legislative machinery which may take precedence over local government decisions is the Victorian Civil and Administrative Tribunal (VCAT). Planning decisions which cannot be resolved between local government, developers and the community can be referred to VCAT for adjudication. VCAT is required to consider the provisions of the State legislation but local government planning frameworks provide guidance only and are not binding. As a result, there is a perception that VCAT is pro-development and against local government. This sentiment was expressed by a programme coordinator:

...when councils want to make changes to their local planning provisions then there are issues with getting that approved through VCAT. So if they want to make changes to their local ones and say "alright with all the new developments that are going to come through they need to have a walking and cycling path and they need to be interconnected" ... developers can then take that to VCAT and say "well actually we're not happy with that, it's costing us money". Quite often at the moment that's [council wishes] been overturned by VCAT

Another way in which State legislation can affect local governments' ability to pursue its own priorities occurs when otherwise innocuous legislation places unexpected downstream obligations upon local government. A strategic planner from local government explains this effect in the context of State Government anti-tobacco legislation prohibiting smoking within bars and clubs requiring local governments to take step towards enforcement and to consider the secondary implications such as street cleaning, etc.:

*The other thing that we find interferes with that strategy is a new requirement by the State Government ... like tobacco legislation. We ended up being required to do the enforcement of new tobacco legislation, which wasn't in our plan, wasn't resourced, but we have to do it, so that means we have to adjust our component of that plan*.

## Discussion

This paper aimed to discover the barriers and facilitators local councils faced when attempting policy and regulatory change to improve environments for physical activity or healthy eating.

We found that councils were more likely to support healthy policy changes that were based on problems with a perceived local evidence base and that could be shown to be cost effective for the council. Some councils found existing local data more relevant to setting health policy than broader data.

Policy change was more likely when supported by external funding although the longer term sustainability and fears of cost-shifting remain concerns for local councils. Regardless of funding local government accepted some mandate to *improve *the health of their community beyond the traditional role of 'protecting' health through sanitation and food safety.

Councils which involved all areas of their organisation in policy development appeared to provide the strongest and most sustainable policy intervention and this was particularly true where council had strong leadership. Other important factors like community lobbying and general resourcing differences within councils also have some impact on the policy direction taken.

It appears that one of the strongest ways to lead policy change to prevent obesity is by presenting an evidence base and business case for local government intervention. Within the Australian Federal system local councils see health policy as the responsibility of State and Federal government and have varying awareness of the role they may have in preventing obesity. Developing the economic case for alleviating the burden of disease on councils will generate local government attention and engagement in this issue.

The development of a 'groundswell' for particular policies to prevent obesity may be another way of creating policy change within local communities and one which also has the potential to grow regardless of council boundaries. Like the example given by one interviewee regarding recycling, our results suggest that councils will regulate to community wishes even in the face of evidence to do otherwise. Currently there appears to be little community agitation for action on obesity. An improved general understanding of the health and economic burden of obesity carried by the community may shift this position.

Any 'groundswell' of community opinion is more likely to be supported by proactive, well resourced councils with expertise in policy change for improved health. The snowball sampling technique used in this research limits participants to those key informants identified by those with in depth knowledge of local government policy. Thus our data may only reflect the views of the leaders in the field who in turn are more likely to represent well resourced and proactive councils. In other councils where there is less local support (and therefore where the need is greatest) state government mandates for change and increased funding provision may play a more important role. It is well established that lower SES areas have the highest levels of obesity. Because of the rate mechanisms that local councils rely on as the primary source of income it is these same low SES areas which have the lowest funding base to work from. This systematic weakness in local government funding means those areas with the greatest health disparities have the least resources to address them. This disparity represents a major structural issue that needs to be addressed by State Government before any realistic improvement in health inequalities could be expected.

State and Federal governments may also need to align their funding schemes to create a uniform approach to healthy eating and physical activity interventions and policy within local government. We have shown that the State government can use funding to set the agenda at local level. Our research suggests that local councils who have structures that allow cross-department policy development have more success in implementing health policy. Within our study we found little desire for such evidence-based multi-level collaborative and strategic approaches. This is a concern as the most effective approach to preventing obesity is likely to involve all tiers of government and be evidence led. Further work is needed to develop collaborative policy approaches which are complimentary across local, state and federal government.

A number of authors have proposed potential regulatory intervention at a local level to prevent obesity. Ashe et al., [26] suggest obesity prevention can be attempted by local regulatory intervention within the school environment, the built environment, by opening up community facilities for broader use, by changing the point of sale environment and through taxation and fees. Others place healthy food policy within a broader call for legislation to create healthy and sustainable communities [27].

There are numerous, although by no means exhaustive, proposals for regulatory intervention to prevent obesity. Very few studies, however, have considered the practicalities of implementing such changes. This study provides valuable insight to previous work by identifying the ways in which such changes might be best supported and why they may be most likely to fail. There has been no work to our knowledge that has bought these two areas together; proposing regulatory change at local government level and testing the feasibility of these changes at local government level.

We have identified that an evidence based 'business case' is the most likely rationale for the support of health promoting policy within local government. It is plausible that cost benefit analyses or other types of burden of disease work from within the academic paradigm may not be helpful nor concise enough for this purpose. Local government needs practical, relevant data at the local government level. Further work is needed to develop an evidence base which can meet the needs of these decision makers and help drive public policy. One practical way of doing this would be for future research to design studies that can provide results at local government level.

Qualitative research can be limited by the smaller study populations when compared with the larger sample populations. One way we attempted to reduce this limitation was by ensuring data saturation had occurred. That is, we continued conducting interviews until we felt no new information was being collected. In addition we interviewed a wide variety of local government experts, including employees of local councils.

Further theoretical work is needed to understand models for potential intervention and to consider potential adverse effects of any regulatory change. Taking a systems approach, for example, would allow us to consider the effect of any intervention across the whole range of council activities [28].

## Conclusion

We found that policy change at local government level is often made in response to evidence about existing problems and the perceived cost benefit of making such change. External funding plays an important part although there are concerns within local government about the ongoing sustainability of policies should external funding be removed. The development of a systematic evidence base to provide clear feedback on the size and scope of the obesity epidemic at a local level, coupled with cost benefit analysis for any potential regulatory intervention, are crucial to developing a regulatory environment which creates the physical and social environment to prevent obesity.

## Competing interests

The authors declare that they have no competing interests.

## Authors' contributions

BL, AP, ML, BAS contributed to the design of the programme of research. SA, EG, and BC conducted interviews and analysed data. All authors contributed to the study design. All authors contributed to the drafting of the original manuscript. All authors contributed to review of the manuscript in light of reviewers comments
